# Median sacral artery injury following a bone marrow biopsy successfully treated with selective trans-arterial embolization: a case report

**DOI:** 10.1186/s13256-016-0827-5

**Published:** 2016-02-24

**Authors:** Yousof Al Zahrani, David Peck

**Affiliations:** Division of Interventional Radiology, Medical Imaging Department, University Hospital, London Health Sciences Center,The University of Western Ontario, London, ON N6H 0B1 Canada

**Keywords:** Bone marrow, Computed tomography, Endovascular treatment/therapy, Hematoma, Iatrogenic injury

## Abstract

**Background:**

Iatrogenic arterial injury during bone marrow biopsy is an extremely rare complication. We present unreported complication of median sacral artery injury that was managed successfully with endovascular treatment.

**Case presentation:**

A 22-year-old Caucasian man known to have end-stage renal disease secondary to Senior-Loken syndrome presented with anemia. He underwent an investigation with bone marrow biopsy that was complicated by hypotension and a further significant drop in his hemoglobin level. Cross-sectional imaging with computed tomography demonstrated a large abdominopelvic retroperitoneal hematoma and active bleeding of the median sacral artery. A successful lifesaving endovascular trans-arterial embolization was performed on an emergency basis and our patient was discharged in a stable condition a few days later.

**Conclusion:**

Iatrogenic arterial injury after a bone marrow biopsy is extremely rare. To the best of our knowledge, a median sacral artery injury has not been previously reported. Endovascular trans-arterial embolization is a safe, effective, and minimally invasive therapeutic option.

## Background

A bone marrow biopsy is a commonly performed diagnostic procedure and is used to establish the diagnosis of a wide variety of benign and malignant hematological disorders. It is also used to evaluate the prognosis and treatment response in certain conditions. It is considered to be a relatively safe procedure. Complications related to this procedure are rare and include local infection, hemorrhage, nerve damage, bone fractures, and needle tract seeding.

A retroperitoneal hematoma secondary to iatrogenic arterial injury is extremely rare and few isolated cases have been reported in the literature. The majority of these cases were due to superior gluteal artery injury. Iatrogenic arterial injuries of the iliolumbar artery, circumflex iliac artery, and hypogastric artery have been reported (Table 1). To the best of our knowledge, none of these cases was due to injury of the median sacral artery.

**Table 1:** Author, patient’s age, bleeding artery, treatment, and outcome of patients with arterial injury following bone marrow biopsy published in the medical literature

Author	Age(years) gender	Bleeding artery	Treatment	Outcome
Arellano-Rodrigo *et al*. [1]	41 male	Circumflex iliac artery	Transcatheter embolization	Completely recovered
Luoni *et al*. [2]	74 female	Iliolumbar artery	Surgery	Completely recovered
Ilhami *et al*. [3]	76 male	Superior gluteal artery	Transcatheter embolization	Completely recovered
Chamisa [4]	29 male	Superior gluteal artery	Transcatheter embolization	Died
Sullivan *et al*. [5]	55 male	Superior gluteal artery	Transcatheter embolization	Completely recovered
Neese *et al*. [6]	67 female	Anterior hypogastric artery	None	Completely recovered

## Case presentation

A 22-year-old Caucasian man with end-stage renal disease secondary to Senior-Loken syndrome presented with high creatinine and low hemoglobin levels. He reported symptoms of fatigue and some shortness of breath with activity. Our patient did not have any history of bleeding, or any evidence of bleeding on physical examination at that time. He had no complaints of chest pain or palpitations, or any postural symptoms.

On examination, our patient looked pale. He had a pulse of 88 beats per minute and a blood pressure of 145/85 mmHg. A head and neck examination did not reveal an elevated jugular venous pressure. A chest examination revealed equal air entry bilaterally, without any adventitious sounds. A cardiovascular examination revealed a normal S1, S2, without any murmurs or adventitious sounds. Abdominal exam revealed a slightly distended abdomen, but it was soft and non-tender with good bowel sounds. Examination of his extremities revealed minimal edema bilaterally.

His laboratory investigation showed a hemoglobin level of 46 g/L and a platelet count of 161 × 10 cells/L. His sodium level was 140 mmol/L, potassium 3.4 mmol/L, chloride 104 mmol/L, bicarbonate 17 mmol/L, urea 33.2 mmol/L, and creatinine 320 μmol/L. His international normalized ratio was 1.0. Our patient was admitted and received two units of packed red blood cells. In order to establish a diagnosis, a bone marrow biopsy was arranged. The procedure was performed using a strict aseptic technique with local anesthesia. A right posterior superior iliac spine approach was used. The biopsy required two attempts before the sample was obtained. The procedure was well tolerated and our patient left the procedure room in a stable condition. A few hours after the procedure, our patient developed pelvic pain and discomfort. His hemoglobin level dropped from 93 to 55 g/L. He then became hypotensive. He was briefly admitted to the intensive care unit for pressor support.

**Fig. 1:**
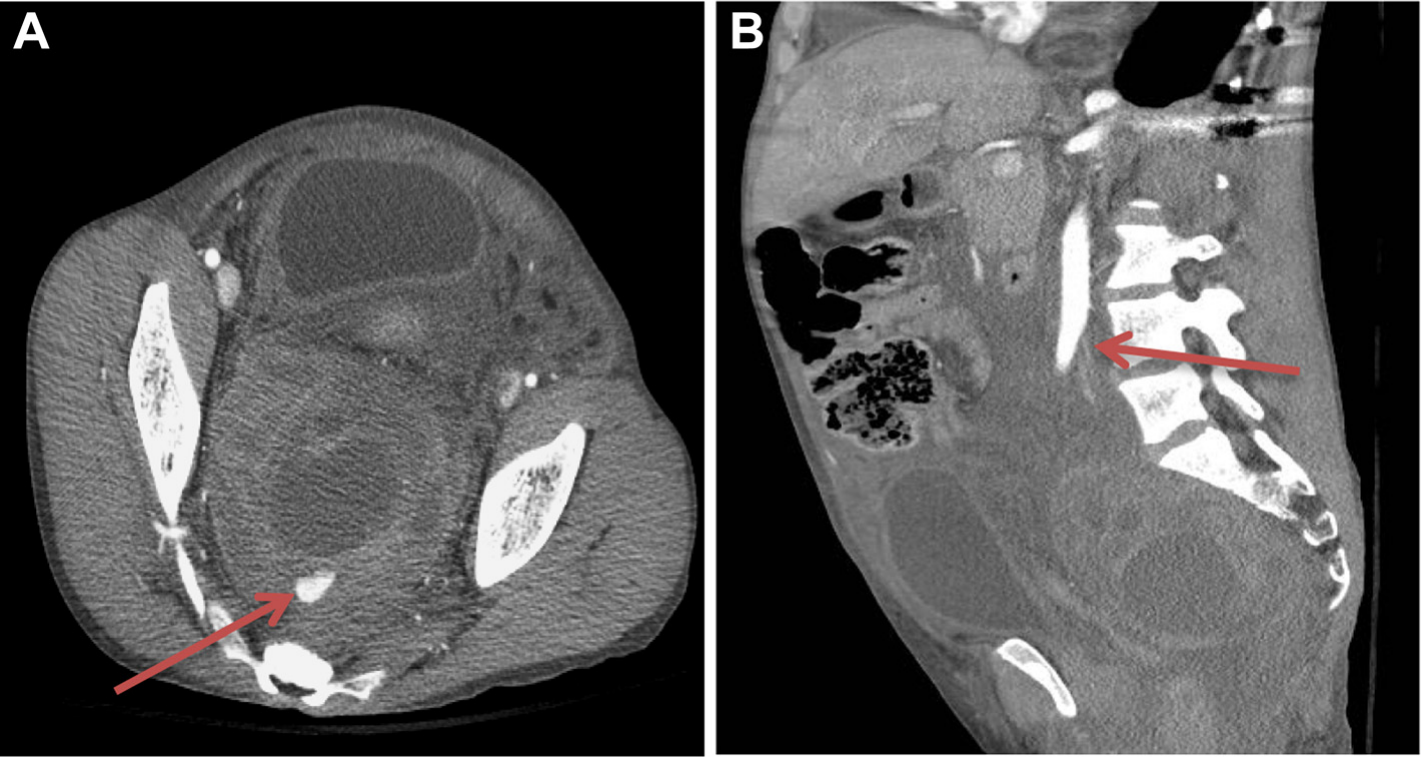
**a** Axial contrast-enhanced computed tomography scan showing a large heterogeneous retroperitoneal/pre-sacral hematoma with active extravasation (arrow). **b** Sagittal contrast-enhanced computed tomography scan showing the origin of the median sacral artery from the distal abdominal aorta (arrow)

A computed tomography (CT) scan of his abdomen and pelvis with intravenous contrast was performed. It showed a large mixed-attenuation retroperitoneal hematoma involving the left psoas, extending into the midline and pelvis. It extended cephalad to the level of his first lumbar vertebra. There was an arterial extravasation in his pelvis, anterior to his inferior sacrum (Fig. 1a). The arterial supply was the medial sacral artery, which arose at the level of the aortic bifurcation (Fig. 1b).

**Fig. 2:**
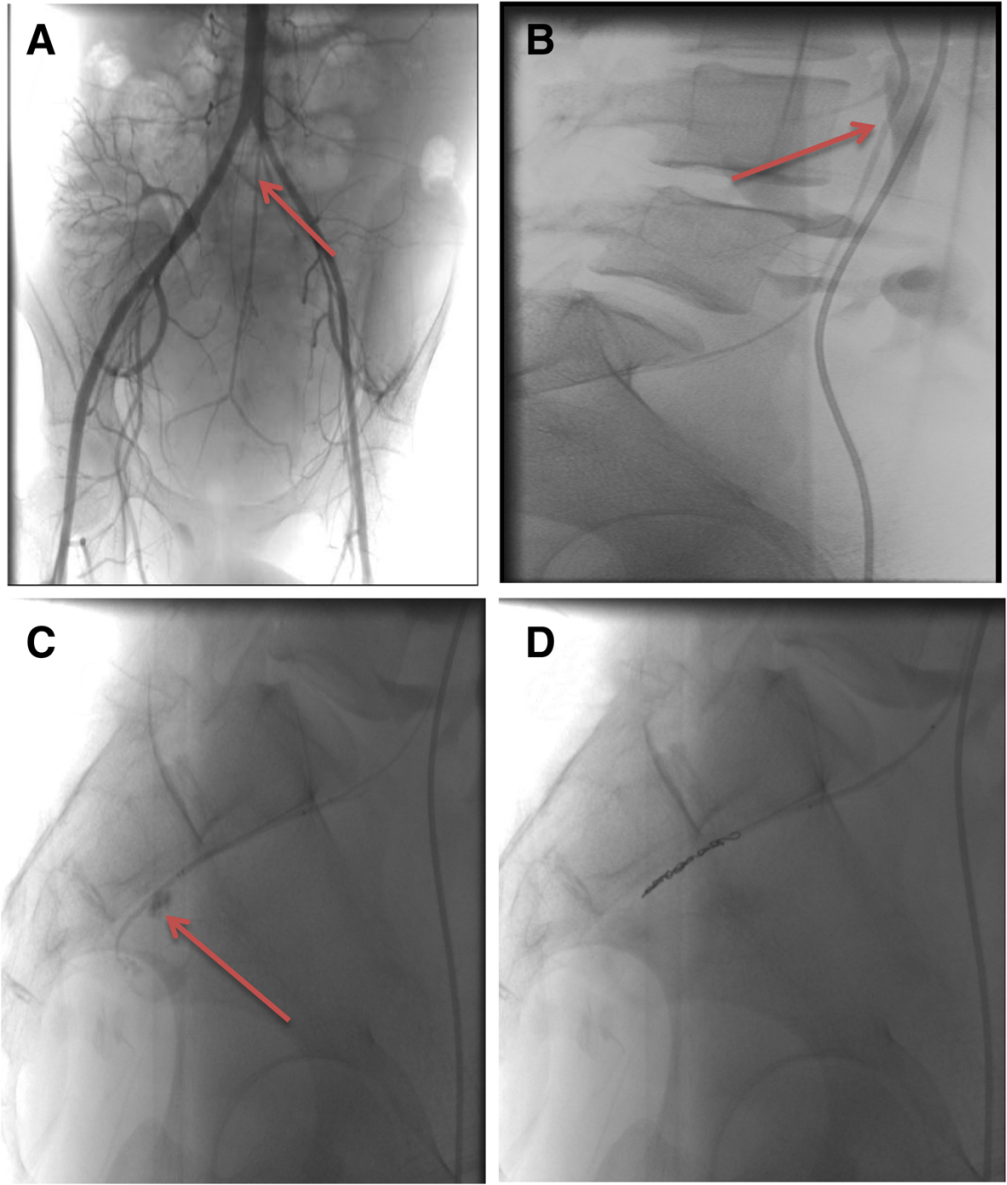
**a** Pelvic angiogram showing the origin of the median sacral artery (arrow). **b** Lateral angiogram image showing the tip of the reverse curve angiographic catheter within the origin of the median sacral artery. **c** Lateral superselective angiogram of the median sacral artery while using the micro-catheter, showing active bleeding (arrow). **d** Lateral angiogram showing multiple micro-coils successfully deployed within the median sacral artery

An emergency angiography and embolization procedure was arranged. His right common femoral artery was punctured in a retrograde fashion using a single wall needle. A 5-French vascular sheath was placed over a guide wire. A 5-French straight flush catheter was advanced through the sheath and his abdominal aorta was selectively catheterized. Three-dimensional rotational pelvic arteriography was performed. No active bleeding was seen on this angiogram (Fig. 2a). Using lateral fluoroscopy, a 5-French reverse curve angiographic catheter was used to engage his median sacral artery ostium (Fig. 2b). Using a combination of a micro-catheter and guide wire, his median sacral artery was selectively catheterized and the micro-catheter combination was advanced into the pre-sacral portion of his median sacral artery. Contrast injection demonstrated active extravasation, as demonstrated on the CT scan (Fig. 2c). A total of seven micro-coils were advanced through the micro-catheter and the feeding median sacral artery was successfully embolized (Fig. 2d). The micro-catheter was retracted and arteriography demonstrated complete occlusion of the feeding artery at the level of the coils with no further extravasation of contrast seen. The vascular sheath was removed and hemostasis was achieved with manual compression.

Following embolization our patient’s hemoglobin levels stabilized. He was briefly intubated for this procedure and was successfully extubated the next day. The bone marrow biopsy showed hypocellular marrow with unremarkable trilineage hematopoiesis. He was discharged home after one week in a stable condition with stable hemoglobin levels. Our patient recovered very well and continued to have follow-up appointments with the nephrology clinic for 2 years after discharge.

## Discussion

Iatrogenic vascular injury and retroperitoneal hematoma following bone marrow biopsy procedures are extremely unusual complications. There are scattered case reports of retroperitoneal hematoma secondary to arterial injury in the literature. Injury of the superior gluteal artery, circumflex iliac artery, internal iliac artery, and iliolumbar artery have been described in different case reports (Table 1). However, to the best of our knowledge, our case represents the first report of iatrogenic laceration of the median sacral artery following a bone marrow biopsy, which was successfully treated with minimally invasive lifesaving endovascular embolization.

Arellano-Rodrigo *et al*. described a case report of a massive retroperitoneal hematoma secondary to injury of the circumflex iliac artery after a bone marrow biopsy. This was successfully embolized using polyvinyl alcohol particles and one coil [1]. Luoni *et al*. described a similar case of retroperitoneal hemorrhage following a bone marrow biopsy that was secondary to injury of the iliolumbar artery. Their case was managed with laparotomy and surgical ligation [2]. Ilhami *et al*. reported an unexpected complication of bone marrow biopsy of an arteriovenous fistula secondary to injury of the superior gluteal artery, for which successful embolization was achieved with coils [3]. Chamisa reported a fatal retroperitoneal hemorrhage caused by damage to the superior gluteal artery. In this case, embolization was unsuccessful. The patient died 2 days after the procedure and surgery was not undertaken because of the patient’s cardiac comorbidity [4]. Sullivan *et al*. described a superior gluteal artery pseudoaneurysm after bone marrow biopsy that resulted in gluteal compartment syndrome. Treatment with coil embolization was performed [5]. Albrecht *et al*. reported a massive retroperitoneal hematoma after bone marrow biopsy. A CT scan showed bleeding that was likely from the hypogastric artery, whereas angiography showed no active bleeding [6].

There are few other case reports of retroperitoneal hematomas following bone marrow biopsies without arterial active extravasation that have been treated conservatively [7, 8]. Edmundo *et al*. reported a case of post bone marrow biopsy gluteal compartment syndrome secondary to injury of a small perforated gluteal artery that was treated surgically [9]. Wan *et al*. reported three cases of retroperitoneal hematoma following bone marrow aspiration. Only one of these cases showed active extravasation on CT scan images. However, the bleeding vessel was not mentioned [10].

An iatrogenic median sacral artery injury is considered to be very rare. Young *et al*. reported a median sacral artery iatrogenic injury during percutaneous mechanical disc compression. It was treated conservatively [11].

## Conclusion

Retroperitoneal hematoma secondary to arterial injury following a bone marrow biopsy is a very unusual complication. However, if such a complication is suspected, CT angiography is recommended to exclude active arterial extravasation and to identify the bleeding artery. Endovascular management with selective trans-arterial embolization offers a less invasive, more accurate, and reliable method to treat these conditions.

## Consent

Written informed consent was obtained from the patient for publication of this case report and any accompanying images. A copy of the written consent is available for review by the Editor-in-Chief of this journal.
